# Developing a cost-estimation model for work-related stress: An absence-based estimation using data from two Italian case studies

**DOI:** 10.5271/sjweh.3948

**Published:** 2021-04-27

**Authors:** Simone Russo, Matteo Ronchetti, Cristina Di Tecco, Antonio Valenti, Aditya Jain, Francesco Saverio Mennini, Stavroula Leka, Sergio Iavicoli

**Affiliations:** Department of Occupational and Environmental Medicine, Epidemiology and Hygiene, Italian Workers’ Compensation Authority (INAIL), Rome, Italy; Nottingham University Business School, Jubilee Campus, Nottingham, UK; Economic Evaluation and HTA (EEHTA) CEIS, Faculty of Economics, University of Rome “Tor Vergata”, Rome, Italy; Institute for Leadership and Management in Health, Kingston University, Kingston Hill Campus, London, UK; Cork University Business School, University College Cork, Cork, Ireland; Centre for Organizational Health and Development, School of Medicine, University of Nottingham, Jubilee Campus, Nottingham, UK

**Keywords:** cost-benefit, cost-effectiveness, economic evaluation, mental health, occupational health, psychosocial risk

## Abstract

**Objectives:**

This paper discusses the development of a cost-estimation model for work-related stress based on psychosocial risk exposure and absence from work. It presents findings from its implementation and evaluation in two organizations in Italy, using national-level tools developed by the Italian Workers’ Compensation Authority (INAIL). It also provides recommendations for the development of similar cost-calculation methods in other countries.

**Methods:**

The cost-estimation model was based on the human capital approach using an indirect cost indicator: loss of productivity due to days of absence attributable to work-related stress. Furthermore, the population attributable fraction (PAF) epidemiological measure was used to calculate the impact of exposure to work-related stress on the basis of data collected through validated tools developed by INAIL and salary cost data.

**Results:**

The developed model was implemented and evaluated in two organizations, the first in healthcare (N=1014) and the second in public administration (N=534). In the first case, it was found that absence related to work-related stress cost the organization €445 000. In the second case, the cost was €360 000.

**Conclusions:**

The proposed model provides an example of how organizations can incorporate well-established indicators associated with work-related stress (eg, various types of absence, psychosocial risk perception, loss of productivity on the basis of salary costs) in a practical way in cost estimations of work-related stress. Such cost estimation can be applied in other countries and organizations to establish the economic and business case of managing work-related stress.

The rapid development and use of information technology, new types of work contracts and work processes, and changes in the workforce composition have brought about many changes in work organization over the last years. A consequence of these developments is an increased prevalence of psychosocial risks, leading to negative consequences on workers’ health, such as work-related stress. Work-related stress has a recognized impact on workers’ health and organizational productivity (1). Several studies have investigated the link between work-related stress and workers’ ill health such as cardiovascular disease (2), musculoskeletal disorders (3), and mental ill health (4, 5).

A number of studies have attempted to calculate the economic burden of psychosocial risks and work-related stress on the basis of direct (healthcare and social security related), and indirect (productivity/loss of earnings related) costs (6–8), which highlight substantial costs for organizations and society as a whole. A World Bank and World Health Organization report estimated that the lost economic output caused by untreated mental disorders globally – as a result of diminished productivity at work, reduced rates of labor participation, and increased welfare payments – amounts to more than 10 billion days of lost work annually, the equivalent of US$1 trillion per year (9). In Europe, the total cost of mental health disorders is €240 billion/per year, of which €136 billion is the cost of reduced productivity including absenteeism and €104 billion is the cost of direct costs such as medical treatment (7). A systematic review of cost-of-illness studies estimated that the cost of work-related stress ranged from US$221 million to upward of US$187 billion across identified studies from different regions of the world; with the projected cost per working person ranging from US$17.79 to upward of US$1211.84. Around 70–90% of these costs were attributed to loss of productivity while 10–30% were attributed to medical treatments. The review also highlighted that the assessment of indirect costs (eg, absence from work, presenteeism, day loss due to staff turnover) is more effective in calculating the cost of work-related stress, irrespective of the estimation approach used (8).

There is evidence that organizations do not necessarily identify costs related to psychosocial risks, as only a relatively small percentage of employers indicate they manage issues such as work-related stress due to a decline in productivity or high absence rates (10, 11). As a result of this, systematic, continuous and strategically aligned psychosocial risk management is scarcely applied in organizations (12). Awareness of the cost of work-related stress can be raised by providing organizations with methodologies that enable them to estimate the cost of work-related stress at the organizational or departmental level. This would act as a driver for organizations to deal with work-related stress in a sustainable manner (7, 10). However, it has been highlighted that attention needs to be paid to how costs and outcomes are measured and valued. For both costs and health-related work productivity outcomes, the measurement tools used for data collection should be clearly reported and the tools valid (13). While some tools/methodologies to help employers establish the costs of poor employee health to their organization and create a business case for taking action have been developed (eg, 14, 15), few can help estimate the cost of work-related stress or exposure to psychosocial risks.

This paper discusses the development of an easy-to-use cost-estimation model for work-related stress (the foundation of a costing tool) based on different types of absence from work and exposure to psychosocial risks. The findings of its implementation and evaluation in two organizations in Italy are also presented.

Studies evaluating the cost of psychosocial risks and work-related stress use two main approaches: a deductive or inductive approach. The deductive approach first calculates the total cost of ill health, and then a percentage estimate of the cases linked to the working activity is applied to obtain the total cost of work-related ill health (8). On the other hand, the inductive approach identifies the different implied costs before calculating and adding them to obtain the total cost of ill health and of work-related ill health in particular (8, 16). The inductive approach generally uses loss of productivity as an indirect cost of ill health and can be used at the national or organizational level to calculate the cost of work-related ill health more accurately (16).

The most commonly used approaches for estimating loss of productivity due to ill health are the friction cost approach (FCA) and the human capital approach (HCA) (17). While both often use the salary as a proxy for calculating productivity costs by multiplying the salary to the hours (or days) lost (18), there is a significant difference in how they estimate costs. FCA counts the number of hours not worked due to ill health until the organization replaces the absent worker, while HCA calculates the lost gross income during the time of absence from work until the worker returns to work or exits the workforce for retirement (19, 20). Accurate estimation of productivity costs remains a highly debated topic, and while estimates of economic burden of chronic conditions are generally much lower when FCA is used, HCA remains the predominant method used to estimate productivity costs (17).

Most studies on the cost of work-related stress have focused on costs associated with absenteeism, presenteeism and turnover (21, 22). While the interplay across such outcomes of exposure to work-related stress should be recognized, it is unlikely that organizations record all cost indicators identified in the literature, and it is therefore important to identify and use those cost indicators that are appropriate, and easy to calculate. Previous studies have suggested that costs associated with absence fulfil these objectives (16).

Absenteeism is the failure to report for work as scheduled, due to involuntary or voluntary factors (22). Organizations have a vested interest in reducing absenteeism since it represents a cost and is directly associated with loss of productivity. Using absence as a cost indicator is also a sensible choice due to its wide use and direct link to loss of earnings that allows good comparability in different contexts. Furthermore, information necessary for cost estimation is often readily available in organizations. This includes the number of working days lost and wage information according to employee position and tenure. In instances where such data is not available within organizations, it is still possible to calculate costs by using estimates of the average hourly wage according to collective labor agreements, broken down by gender, occupational position, age and other occupational characteristics. Therefore, taking into account the difficulty in identifying and/or quantifying different kinds of existing costs related to work-related stress (8, 16) and the need to select cost indicators that can be easily collected by organizations, this study focuses on absence from work as an indirect cost of exposure to psychosocial risk and work-related stress.

## Method

### Procedure and measures

Risk assessment for work-related stress has been a legal obligation in Italy since 2008. It should be noted here that legal requirements specify that employers should assess ‘work-related stress risk’ to refer to psychosocial risk (23). In line with this, we will use the terms ‘work-related stress risk’ and ‘work-related stress risk assessment’ in this paper to refer to psychosocial risk and psychosocial risk assessment.

According to national legal requirements, as a first step in the risk assessment process, organizations must consider objective indicators and data records (such as injuries, sick leave, turnover rate) as potential signs of the impact of work-related stress. In addition, they need to identify psychosocial hazards that might be negatively affecting specific work groups or the working population in the organization. Findings from this preliminary assessment lead to the implementation of preliminary measures to manage the emerging psychosocial risk areas. If these measures do not improve the situation sufficiently, organizations must proceed to conduct a further in-depth work-related stress risk assessment based on employee perceptions.

The main methodological approach used for the assessment and management of work-related stress risk in Italy is a methodology developed by the Italian Workers’ Compensation Authority (24), which uses two main tools. First, a checklist is used for the preliminary assessment, which includes objective indicators associated with work-related stress as evidenced in the literature such as work-related injuries, sick leave absence, other absence from work, left over vacation days, turnover, legal action/disciplinary sanctions, formal records of employees’ complaints to the company or to the company’s occupational physician (25). This information is collected from organizational records by a Steering Group that includes the employer or his/her representative, a health and safety professional working for the organization, the occupational physician and the employee representatives. In addition, the second part of the checklist is used to identify work-related stress risks on the basis of group discussions with workers at unit level (referring to homogenous groups^1^ of workers), that have specific work-related risk factors and organizational aspects in common (23).

Second, for the in-depth assessment of work-related stress risk, a validated questionnaire, the Management Standards Indicator Tool (MS-IT), an adapted version of the UK tool, is used (26, 27). This tool enables organizations to assess employee perceptions of psychosocial risk factors and is in line with good practice recommended by the European Framework for Psychosocial Risk Management (PRIMA-EF) (28). This multi-layered method of data collection offers important opportunities for the identification of costs associated with work-related stress since it drives organizations to collect data that can also be used for cost estimation purposes.

### Development of the cost-estimation model

We developed a cost-estimation model of work-related stress based on one of the most widely used indirect cost indicators – loss of productivity due to days of absence attributable to work-related stress using the HCA. One of the main challenges with using an inductive approach such as HCA is related to the weight assigned to the different implied cost components in order to identify the real economic burden of ill health (29). In the case of work-related stress, it is difficult to estimate the extent to which the days lost due to sickness absence are directly due to work-related stress. Several studies report figures based on the calculation of an “attributable fraction”, ie, the part of a negative outcome (for example sick leave) calculated as attributable to the exposure to psychosocial risk and work-related stress, which is a measure of context. This method allows obtaining the costs related to work-related stress from the total financial burden associated with that negative outcome (eg, sick leave) (6). In light of this and following Bejean & Sultan- Taieb’s recommendations (6), the following formula was developed for calculating the cost estimation of work-related stress (*Cost w.r.s.t*):


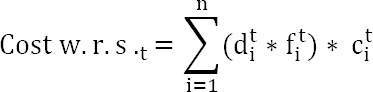


where *d^t^_i_* are the number of days of absence from work due to injuries, sickness, and other reasons such as extended leave for personal reasons and unauthorized absence in the year *t* for the homogenous group *i*. The *c^t^_i_* is the average cost of a working day for the year *t* in the homogenous group *i*. The *f^t^_i_* is the average fraction or percentage attributable to work-related stress risk.

#### Days of absence from work

Days of absence from work was further sub-divided into:

1. Days of absence due to injury at work;
2. Days of absence due to sickness;
3. Days of absence due to other reasons, such as extended leave for personal reasons, unauthorized absence.

In the proposed formula, the days of absence are calculated at the homogenous group level. In order to assess the average cost of a working day (or the selected unit of time), it is possible to use different parameters. The best parameter identified is the worker’s income per unit of time considered. However, in case such data is not available for each worker, it is possible to consider the average income by professional category within the company or at national level. Accordingly, since income per unit of time for the single workers was not available in the two case studies considered, we decided to apply to the workers the average salary relative to their professional categories that were identified by the two organizations. Then, the cost of total days of absence by homogenous group (*A. costi*) was calculated by the following formula:


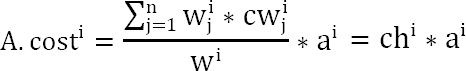


where *w^i^_j_* is the number of workers with job *j* in the homogenous group *i*; *w^i^* is the number of workers in the homogenous group *i*; *cw^i^_j_* is the estimated average cost of a working day for a worker with professional category *j* in the homogenous group *i*; *a^i^* is the total number of days of absence in the homogenous group *i*; *ch^i^* is the estimated average cost of a working day of the homogenous group *i*.

#### Work related stress attributable fraction

Absence from work has concurrent determinants, but, in this study, we were interested in calculating the potential impact of work-related stress on the number of absence days from work for each homogenous group. According to the literature, this could be done using an attributable fraction, as an epidemiological measure generally used to calculate the contribution of a risk factor to a specific disease (30). The general formula used for calculating the population attributable fraction (PAF) (30) is reported as follows to show how we proceeded in adapting this to estimate the contribution of work-related stress to absence from work:


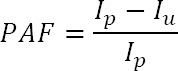


where *I_p_* is the incidence in the population and *I_u_* is the incidence in the unexposed population.

Starting from the general PAF formula, we proceeded in adapting this to develop a work-related stress attributable fraction formula (*W.r.s.at.fract.i*), by considering the incidence in the population as the number of days of absence (measured with the three indicators included in this study) for each homogenous group, and the work-related stress risk as the exposure factor:


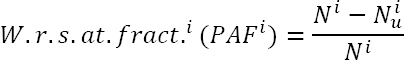


where *N^i^* is the total number of absence days from work in the homogenous group *i* and *N_u_^i^* is the number of absence days from work for unexposed workers to work-related stress risk in the homogenous group *i*.

To apply the work-related stress attributable fraction formula to our data, we also needed to calculate the number of unexposed workers to work-related stress risk. To this aim, we used the scores obtained from workers by filling in the MS-IT, a work-related stress questionnaire of 35 items that measure seven psychosocial hazard dimensions (demands, control, management support, colleague support, role, relationships and change). Higher scores obtained by the questionnaire generally reflect better working conditions (ie, a more positive psychosocial work environment). In order to identify those workers that can be considered unexposed to the work-related stress risk, threshold values need to be considered and these are provided through the questionnaire for each of the seven dimensions, based on a large normative national sample. In our study, it was necessary to calculate a unique score for our estimation model as a general measure of work-related stress risk. Thus, we used national data from INAIL’s web platform consisting of 66 118 questionnaires collected from different organizational settings and uploaded at the time of the study. Using a distributive criterion, we defined four risk groups (high <20%, medium-high <50%, medium-low ≥50%, low >80% risk) measuring the threshold values and related quartiles from the general distribution of INAIL’s national database. Those with higher scores than the ones observed in the first quartile were classified as unexposed workers to work-related stress risk, and accordingly those with lower scores were classified as exposed workers. Thus, we applied the threshold values calculated on the national dataset to our study to identify the unexposed workers in each homogenous group based on their scores on the MS-IT. Then, we verified the frequency of absence days from work of unexposed workers (*N_u_^i^*) with respect to the total number of absence days from work in the homogenous group (*N^i^*). Finally, we applied the work-related stress attributable fraction formula (*W.r.s.at.fract.i (PAF^i^)*), by subtracting the number of absence days from work reported by unexposed workers in a specific homogenous group (*N_u_^i^)* from the total number of absence days from work in the same homogenous group (*N^i^*). In this way, we obtained a weighted measure of the impact of work-related stress risk on the number of days of absence from work for each homogenous group (i), namely the work-related stress risk attributable fraction (PAF*^i^*).

### The case studies

Two case studies were selected among the organizations using the INAIL methodology to test the proposed cost-estimation model of work-related stress risk. The selection criteria of the case studies were: (i) the availability of data through the application of both phases of INAIL’s methodology (the checklist and the MS-IT); and (ii) being an organization in two high risk sectors for work-related stress in Italy: healthcare and public administration (31).

The first case study was a public hospital where data was collected on 14 homogenous groups of healthcare workers (N=1014). The second case study was a public administration department, where data was collected on 6 homogenous groups of workers (N=534). Objective indicators of days of absence from work were extracted in both of these organizations for each homogenous group using data records that were obtained through the use of the checklist for the preliminary assessment of work-related stress risk. All the workers belonging to the homogenous groups were also included in the in-depth assessment conducted through the use of the MS-IT. Responses were matched to the respective homogenous group, which enabled the identification of the number of exposed/unexposed workers to work-related stress risk for each homogenous group by applying the cut-off score extracted by the national sample.

## Results

### Case study 1

In the first case study, 14 homogenous groups of healthcare workers from a public hospital were included where preliminary and in-depth work-related stress risk assessments were conducted using the INAIL methodology in 2018. To estimate the cost associated with absence for each homogenous group, we were able to link absence to the job positions of workers in collaboration with the organization and calculated the average cost of a working day per position using data published on the hospital website (table 1). The average annual cost per single worker is the total yearly average cost per type of occupational position divided by the number of workers. The monthly average cost is the annual average cost per single worker divided by 14^2^. Finally, the monthly average cost of a worker divided by the average number of working days in a month (working days in a year/months of a year) estimates the average cost of a working day per single worker (*c^t^_i_*).

**Table 1:** Workforce of the hospital and cost estimate of working days.

	Workforce				Cost estimate			
	
	Permanent	Fixed term	Total	Staff cost (€)	Yearly cost / worker (€)	Monthly cost 14 mth pay (€)	Working days	Cost of a working day (€)
Management staff, physicians	294	7	301	26 822 757	89 112	6365	253	301.90
Health staff	435	11	446	18 917 827	42 417	3030	253	143.70
Management staff, other jobs	10	1	11	1 062 772	96 616	6901	253	327.30
Staff, other jobs	255	1	256	8 579 999	33 516	2394	253	113.50

To provide an in-depth explanation of how costs were estimated for each group, table 2 presents an example of one homogenous group (Reconstructive plastic surgery). In this example, the number of workers per type of job position, the related percentage and the average cost of a working day are reported. The average cost of a working day in this group was calculated using the formula for calculating the average cost of total days of absence by homogenous group (€183). Then, the cost associated with work-related stress risk (*Cost w.r.s.t*); €177 538) was calculated by applying the specific attributable fraction of the group (*f^t^_i_*; 64.8%) to the total cost of absence (€259 559). This cost was obtained from the product between the total number of absences (*d^t^_i_* 1417) and the average cost of a working day (*c^t^_i_*).

**Table 2:** Estimate of costs associated with work-related stress risk for hospital group Reconstructive Plastic Surgery.

Jobs (N)	Jobs (%)	Cost of a working day (€)	Homogenous group data for costs estimation						

			Injuries	Diseases	Other absences	Total absences	Cost of absence (€)	PAF (%)	Work-related stress risk (€)
Medical director	7	28	301.90	72	272 1073	1417	259559	0.68	177539
Nurse	14	56	143.70						
Healthcare social worker	1	4	113.50						
Care technician	1	4	113.50						
Healthcare social assistant	2	8	113.50						
Total	25	100	183.20 ^a^						

a Average cost.

The calculation was applied to all the homogenous groups included in this study, as presented in table 3.

**Table 3:** Days of absence, attributable fraction for work-related stress risk and cost estimate for all homogenous groups: **Hospital**.

Homogenous group	Absence due to injuries	Sick leave	Other absence	Total absences	Response rate (%)	Absence cost (€)	PAF (%)	Work-related stress risk costs (€)
Reconstructive Plastic Surgery	72	272	1073	1417	76	259559	47.4	122949
Anatomy and Pathological Histology and	13	107	1076	1196	95.7	233 749	31.8	74 375
Cytodiagnostic								
Technical unit and Clinical Engineering	17	336	919	1272	80	144433	33.3	48 144
Hematology	0	14	610	624	94.1	124513	37.5	46 692
Orthopaedics	0	100	576	676	81.3	115 922	30.8	35 668
Laboratory of Medical Physics and Expert Systems	0	156	748	904	81.3	102 647	30.8	31 584
Derma pathology	0	68	343	411	75	81 896	33.3	27 299
Insurance, Litigation and Deliberative Acts	0	2	331	333	85.7	37 811	50.0	18 906
Cardiology	0	113	777	890	78.6	188239	9.1	17 113
Endocrinology	0	10	370	380	85.7	80 372	16.7	13 395
Training	0	72	363	435	85.7	51 267	16.7	8 545
Respiratory Pathophysiology	0	39	419	458	88.9	94 950	0.0	0.0
Cancer Dermatology	0	36	539	575	90.9	129096	0.0	0.0
Digestive surgical oncology	0	94	559	653	100	129327	0.0	0.0
Total/Average	102	1419	8703	10 224	85.6	1 773780	24.1	444 669
Weighted average/costs							25.9	41 781

Overall, an estimated 10 000 days of absence were calculated, costing the organization about €1.8 million. Absence related to work-related stress risk cost the organization about €445 000 (24% of the absence cost). The weighted average (taking into account the different number of questionnaires per group) was found to be about €41 780, with a standard deviation of €33 900. Three homogenous groups (Insurance and Litigation and Deliberative Acts, Reconstructive Plastic Surgery, Hematology) reported an attributable fraction higher than 35%, while five groups reported an estimated attributable fraction of 30–35%, and six groups an estimated fraction value <20%. The Reconstructive Plastic Surgery group reported the highest cost (€123 000) due to work-related stress risk, with an attributable fraction equal to 24.1% and 10 224 absences totally, corresponding to 1.6 times the estimate of the second group with the highest costs (Anatomy and Histopathology and Cytodiagnostics, with an estimated cost of about €74 000). Fifty-five percent of the total estimated cost associated with work-related stress risk corresponds to the first three homogenous groups (33% of the total questionnaires considered).

### Case study 2

The second case study was carried out in a public administration unit and included six homogenous groups. The same data records related to absence from work (absence due to injuries, sick leave, and other absence from work) were taken into account (*d^t^_i_*), to estimate the average cost linked to work-related stress risk for the groups considered (*Cost w.r.s._t_*). Data was extracted for each homogenous group from the preliminary and in-depth work-related stress risk assessments. As in the first case study presented, we calculated the attributable fraction of the cost indicators for each homogenous group *(f^t^_i_)* using the cut-off identified in the national dataset of workers that responded to the MS-IT. Then, the formula for calculating the PAF for each group was applied. In line with case study 1, only those homogenous groups with a workers’ response rate to MS-IT ≥75% were included.

However, in contrast to the previous case study, we could use the monthly salaries as the basis for estimating the average cost of a working day (*c^t^_i_*). Findings reported in table 4 indicate that the estimated average value of the attributable fraction is far from the value observed in the previous case study (25.1%). Furthermore, the second case study reports lower internal variance (standard deviation of 6.5%) compared to the first case study (standard deviation of 17.1%). The six homogenous groups reported 26 564 absences (60% for sick leave), leading to a total cost of about €2 million; 16.2% of this (€360 000) was estimated to be the cost of absence generated by workers exposed to work-related stress risk, while the weighted average per homogenous group was about €220 400 (standard deviation of €151 100).

**Table 4:** Days of absence, attributable fraction by work-related stress risk and cost estimate per homogenous group: **Public administration unit.**

Homogenous group	Absence due to injuries	Sick leave	Other absence	Total absences	Response rate (%)	Absence cost (€)	PAF (%)	Work-related stress risk cost (€)
Civil protection	0	291	0	291	75.4	26 003	10.2	2653
Transportation	0	536	356	892	78.5	85 595	8.1	6903
Regional agency of river basin district	0	378	337	715	83.9	70 531	23.1	16 276
Social policies	0	882	349	1231	81.4	131 626	14.3	18 804
General affairs and information society	2	896	410	1308	83.1	139 086	23.7	33 004
Forest department and environmental surveillance	49	13 083	8 995	22 127	83.0	1 562635	18.1	282404
Total/average	51	16 066	10 447	26 564	80.9	2015478	16.2	360 044
Weighted average/costs							16.4	330 538

## Discussion

Even though there are studies providing national and supranational estimations of the cost of work-related stress and psychosocial risks (8), there are a lack of tools and models that allow organizations to evaluate their economic burden in their own context. Current cost of illness studies on work-related stress and psychosocial risks use various methods to calculate costs and show limitations in terms of the high number of variables considered, costs that are hard to calculate, and lack of availability of information (13, 18).

In developing our cost-estimation model, we used the HCA (19, 20) and focused on an indirect cost of work-related stress, namely loss of productivity associated with absence from work. As seen in both case studies used in this research, absence data were easy to obtain and it was possible to aggregate them at the unit level (homogenous group). Moreover, absence from work data can easily be linked to an economic value by using the salary as a proxy of productivity (8).

Even though sickness absence emerged as the most commonly used indirect cost indicator in the literature, we also included different types of absence in our cost-estimation model to enable organizations to account for all costs associated with loss of productivity due to absenteeism. However, to account for uncertainty in relation to the absence from work that is related to work-related stress, we estimated the level of absence that might be attributable to work-related stress risk by developing an attributable fraction of each absence indicator to the specific exposure factor, using the MS-IT scores). This questionnaire is included in the INAIL methodology (24), which is the most widely used methodology in Italy for meeting legal requirements for assessing work-related stress risk. In other national/ regional contexts, other instruments, where available, can also be used to identify the attributable fraction by using the method discussed in this paper, as long as they cover the required parameters in the proposed model. It is, therefore, recommended that further research evaluates the model in low risk sectors and in other countries where it is possible to use similar tools and parameters. Furthermore, our study did not consider other aspects associated with loss of productivity such as turnover and presenteeism. Such measures could be included in cost-estimation models where available to improve their accuracy. On the other hand, even though the HPA approach used in this study is the predominant method used to estimate productivity costs, it is important to acknowledge that other methods, such as FCA, generally produce much lower estimates of economic burden of chronic conditions (17). Other issues to be considered in future research are the time frames used in cost estimates at national level such as adjusting for timing and uncertainty (eg, 32).

The introduction of new regulation on work-related stress risk assessment in Italy and the development of practical work-related stress risk assessment tools have had a positive impact both in terms of awareness and practice in Italian organizations (33). The INAIL methodology has been made publicly available to Italian organizations and the collection of data at national level through the INAIL platform allows the development of national benchmarks by using cut-off points and applying appropriate weighting (as described in this paper). The developed cost-estimation model is an additional tool publicly available to Italian organizations aiming to further engage them in implementing good practice.

Similar policy contexts to Italy are also found in several countries around the world, particularly in Europe (eg, Belgium, Czech Republic, Germany, the Netherlands, the UK) and other regions (e.g. Australia, Chile, Canada, and Mexico) (1). The cost model developed in this paper can inform the development of similar national benchmarking systems and costing models in countries where policies exist or where national level tools are available. For instance, in the US, a tool will soon be launched by NIOSH in relation to their total worker health (TWH) programme (34). The proposed cost-estimation model could be used to calculate the cost of work-related stress in organizations in conjunction with TWH national level data. While it is acknowledged that the development of such tools requires both strong commitment and investment of resources at country or sectoral level, it is possible to learn from good practice examples that are now available and adapt existing models in new national contexts (35).

Indeed, there is a need to develop further tools based on this method to improve awareness of the cost of work-related stress among employers since the business case and especially the cost of absence have consistently been identified in the literature as key drivers that engage organizations in psychosocial risk management (eg, 12, 13). Such cost estimation tools will supplement other economic evaluation approaches (ie, cost-benefit analysis, cost-effectiveness analysis or cost-utility analysis), which are already used at the organizational level to evaluate the return-on-investment of interventions (13, 36), and provide a direct assessment of their impact on the bottom line (37). The proposed model will also help answer calls for economic evaluation of interventions based on established guidelines and validated consistent measures of productivity costs as the main cost driver (38).

### Concluding remarks

This paper offers a cost-estimation model for work-related stress based on absence and psychosocial risk exposure. The proposed model provides an example of how well-established indicators associated with work-related stress (eg, various types of absence, psychosocial risk perception, loss of productivity on the basis of salary costs) can be incorporated in a practical way in cost estimations of work-related stress by organizations. A key driver for the protection and promotion of health and well-being at work is the business case which focuses on the notion of financial costs, as well as benefits for organizations. Since all organizations require workers in order to achieve their goals, there is a strong business case to be made for ensuring that workers are mentally healthy through occupational health and safety management (37). The cost-estimation model proposed in this paper provides the starting point for developing such a business case. However, it can also be useful towards developing a more holistic ‘value case’ that also accounts for intangible business benefits associated with mental health and well-being at work (39). The ‘value case’ can help organizations internalize the value of addressing issues such as psychosocial risks and work-related stress and incorporate them in all organizational strategies, systems, and behaviors, therefore moving towards sustainable good practice. The need for a holistic approach is particularly important as not only financial reasons, but also legal and moral reasons drive organizations to manage psychosocial risks and promote health and well-being at work (40). The cost-estimation model proposed in this paper provides the starting point for developing such a value case and sustainable good practice.

### Conflict of interest

The authors declare no conflicts of interest.

## Footnotes

1 The focus of the assessment are the homogenous groups, namely groups of workers sharing common features related to both the job and the context (in terms of job design, goals, procedures, management and communication styles, resources, relationships, and support from colleagues and direct supervisors) that are the potentiala sources of stress.

2 In Italy 14 months per year are paid for each worker.
